# Sleep-wake cycle impairment adding on the risk for COVID-19 severity in people with diabetes

**DOI:** 10.5935/1984-0063.20200038

**Published:** 2020

**Authors:** Mark Thomaz Ugliara Barone, Belinda Ngongo, Luiz Menna-Barreto

**Affiliations:** 1 International Diabetes Federation (IDF), Board of Directors - Brussels - Brussels - Belgium.; 2 ADJ Diabetes Brasil, Research and Education - Sao Paulo - SP - Brazil.; 3 Fórum Intersetorial para Combate às DCNTs no Brasil (FórumDCNTs), General Management - Sao Paulo - SP - Brazil.; 4 Sociedade Brasileira de Diabetes (SBD), Education - Sao Paulo - SP - Brazil.; 5 Pan African Women in Health (PAWH), Founder - Johannesburg - Gauteng - South Africa.; 6 Escola de Artes, Ciências e Humanidades - Universidade de Sao Paulo (EACH-USP), Grupo Multidisciplinar de Desenvolvimento e Ritmos Biológicos (GMDRB) - Sao Paulo - SP - Brazil.

**Keywords:** Diabetes Mellitus, Coronavirus Infections, Pandemics, Circadian Rhythm, Sleep Disorders, Circadian Rhythm, SARS Virus

## Abstract

In the present article, we explore the risks of circadian disruptions and impact on the sleep-wake cycle of individuals with diabetes during COVID-19 pandemic. The association between the duration and quality of sleep and the stability of glucose levels is well-established. Therefore, during the pandemic with changes and limitations in the exposure to cyclic cues that entrain the circadian rhythms, such as light-dark and social interactions, we hypothesize that the power and stability of circadian rhythms decrease if measures are not taken to intentionally create a routine that includes zeitgebers. Knowing that sleep-wake cycle disruptions impair melatonin production, immune system response and glucose metabolism, and that individuals with diabetes are at higher risk for poor prognosis when infected by SARS-CoV-2 (especially if their blood glucose is out of target), we recommend monitoring and advising these individuals towards strategies to maintain adequate sleep quality and duration as part of their preventive and protective measures during the new pandemic routine.

## INTRODUCTION

In early 2020, COVID-19 rapidly became the main global health concern due to its swift spreading pandemic characteristics. Reports from the different epicenters revealed severity and mortality of this novel virus more pronounced in men and elderly populations and in individuals with non-communicable diseases (NCDs), especially hypertension, diabetes, obesity, cancer and pulmonary chronic conditions^1-3^. Hygiene measures, social or physical distancing, and stay-at-home recommendations were established as main strategies to reduce transmission and to allow health systems preparedness in anticipation for the surge in demand in COVID-19 related services^4,5,6^. These interventions would also help to protect the population whilst allowing the recovery of those infected. While this seems reasonable from an epidemiological perspective, from a socio-economic-biological point of view, new challenges arose, including those with the potential to hamper the continuous self-care of individuals with diabetes and other NCDs^1^.

Whereas, in China, the overall case-fatality rate was 2.3%, it was 7.3% for individuals with diabetes^2^. In Italy, type 2 diabetes was the second most prevalent NCD associated with deaths (31.8%)^1^. Other countries reported similar trends; high severity and mortality of individuals with diabetes and other NCDs when infected by SARS-CoV-2^1,3,7^. Recent studies revealed that more than diabetes, high blood glucose is a predictor of poor COVID- 19’s outcome^7^. Zhu et al.^7^ compared two groups of individuals with type 2 diabetes and COVID-19, one with blood glucose 70-180mg/dL and the other with blood glucose >180mg/dL, and found a lower all-cause mortality rate in the first group (HR=0.14; survival of 98.9% *vs* 89%)^7^. In hospitals in the US, while 41.7% of individuals with hyperglycemia (glycemia >180mg/dL) died, 85.2% of the ones with diabetes and glycemia below 180mg/dL survived (*p*<0.001)^8^. There seems to be various reasons for these outcomes associated with hyperglycemia, ranging from exacerbated expression of the coronavirus entry receptor (angiotensin-converting enzyme 2 - ACE2) to suboptimal immune response and induction of virus replication^7,8,9^.

Being aware of the delicate balance needed for individuals with diabetes to keep their blood glucose on target and knowing that in 62% of the countries services to treat diabetes and its complications were disrupted during the pandemic^10^, we intend in the present article to bring a contribution from chronobiology to potentially reduce metabolic consequences caused by alterations in these individuals’ routine. As widely studied, environmental cycles - especially the light-dark and the temperature - and the social interactions - from the alarm clock ringing, to meals, TV and exercise times - are zeitgebers, i.e., they entrain the endogenous rhythms in a period of approximately 24 hours, regulating the different body systems^11^. This circadian entrainment, leading to synchronized endogenous rhythms, is known for preventing diseases and promoting well-being^6,11^. For this reason, the impact of partial or full lockdown with restrictions in mobility changes the exposure to environmental and social cues, potentially leading to misalignment with environmental cycles and internal desynchrony^6^. Consequently, immune responses become impaired, and metabolic and mental disorders may develop or exacerbate^12-17^. In individuals with either main types 1 or 2 of diabetes, the described consequences of this misalignment, desynchrony or sleep impairment is insulin resistance, leading to increased glycemic levels and variability^6,12,13,16,18,19^.

The new routines varied widely during the pandemic. Few, if any, were able to maintain their pre-pandemic daily life pattern. While some lost their jobs or lacked access to schooling, others, including many healthcare professionals (HCP), worked day and night. Nevertheless, regarding the sleep-wake cycle, those who prior to the pandemic worked during hours that collided with their biopsychological timing might have appreciated waking up later or having a naptime after lunch. Whereas some may experience the feeling of “I can finally sleep the number of hours that I need and feel well”, many others might be “lost in time”^20^, sleeping or waking up at a different time each day, experiencing low sleep quality, fragmentation or insomnia, headaches and humor instability^6,15,21,22^.

Different aspects contribute towards this reduction in the sleep-wake cycle stability; some would be attributed to the psychological uncertainty of life during the pandemic, while others are associated with desynchrony due to limited or irregular exposure to zeitgebers^^6^,^21^,^22^^. In addition to the effect of desynchrony and circadian misalignment decreasing glucose tolerance and insulin sensitivity^^16^,^18^^, individuals with any of the main diabetes types present an additional concern related to reduced production of the night phase hormone, melatonin^^23^,^24^^. Melatonin is known for acting as an endogenous zeitgeber, entraining the different tissues in a circadian pattern, in addition to its powerful antioxidant and immunomodulatory characteristics^^25^,^26^^. As a result, it synchronizes the immune system through its cyclic release, and modulates the innate immunity, through its redox regulation and oxidative stress suppression^^14^,^25^,^26^^. This is understood as fundamental for an optimal response to viruses and other infections^^14^,^15^,^26^^. Therefore, while individuals with diabetes present a decrease in melatonin production associated with blood glucose levels^^23^,^24^^, it is hypothesized that melatonin would protect against COVID-19^^26^^.

Melatonin is intimately associated with the sleep-wake cycle regulation^^25^^. During the pandemic, certain factors may inhibit melatonin production and superficialize sleep. Among them are increased time watching TV and surfing the internet^^27^^ - screen time is negatively correlated with sleep duration and well-being, with screen light being a melatonin and sleep inhibitor^^28^^, and increased glycemia in individuals with diabetes^^27^^. Summing to this scenario of sleep-wake cycle instability the reduction in physical activity^^27^^, a known zeitgeber and traditional pillar for glycemic control in people with diabetes, and the inability of many nations to provide adequate health services and medicines to people with diabetes^^10^,^27^,^29^^, it is not surprising the consequences on their glycemic control^^12^,^27^^. In Brazil, 59.4% of the individuals with diabetes were unable to maintain their blood glucose at the same levels or stability as prior to the COVID-19 pandemic^^27^^. Thus, we see clear reasons for flagging the sleep-wake cycle as a point of attention with the potential to intervene, despite the fact that, to our knowledge, no study was conducted so far to evaluate it in individuals with diabetes during the COVID-19 pandemic.

**Figure 1:**
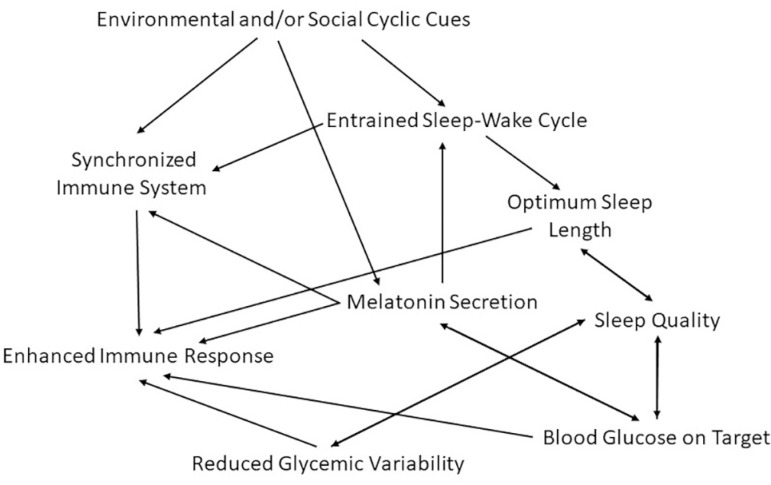
Map connecting environmental and social cyclic cues with the sleep- wake cycle to an enhanced immune response in individuals with diabetes.

Taking into consideration that: 1) COVID-19 severity and mortality in people with diabetes are higher than in people without NCDs, and even higher when associated with increased blood glucose levels^^7^-^9^^; and, 2) misalignment with exogenous cycles during the pandemic may impair individuals’ sleep wake-cycle in different ways, contributing towards the reported inability to keep blood glucose control during the pandemic in people with diabetes^^12^,^13^,^16^,^18^,^19^,^27^^, we see opportunity for contributions from chronobiology and sleep sciences. While it is true that healthcare systems adjustments for access to medicines and healthcare services must be optimized^^1^,^3^,^10^,^27^,^29^,^30^^, strategies of exposure to zeitgebers and sleep hygiene can be widely adopted to assist in achieving the goal of bringing individuals’ blood glucose into target during the COVID-19 pandemic and, this way, improve their immune response and condition to fight SARS-CoV-2 and other potential infections^^7^,^8^,^11^,^14^,^25^,^26^^, as depicted in Figure 1.

## CONCLUSION

Aspects discussed above delineate the consequences of circadian disruption in the population with diabetes, which would make them more vulnerable to severe SARS-CoV-2 infection. Our chronobiological recommendations include measures to entrain circadian rhythms of these individuals and their caretakers, and hence improve well-being, prevent sleep disorders, enhance immune response and aid in maintaining blood glucose levels on target (Figure 1). They should include strategies of exposure to zeitgebers and establishment of a routine that fits individual’s sleep needs including morningness/eveningness preferences, duration and quality. An option to identify the preferable routine would be by filling daily notes (diaries) in which the person captures the time of his/her activities along with evaluations of how he/she feels (mood, motivation and disposition). Doing that for a sequence of several days, would aid the subject to adopt the best routine for his/her well-being.

Strategies of exposure to environmental and social zeitgebers, whilst building a routine, should also be adopted by HCPs whom, in addition to close exposure to the virus, are at higher risk due to immune response impairment caused by shiftwork and exhaustive routines during the pandemic^^15^,^22^^. The recommendations do not necessarily mean a strict agenda, but rather improve the sleep-wake cycle power and stability, while reducing insomnia, short and fragmented sleep, and enhancing melatonin production. The plan must include cyclic elements such as: 1) regular schedule to go to bed and wake up; 2) meals together with home cohabitants; 3) regular online or phone interaction with friends and family; 4) exposure to sunlight in the morning; 5) limited screen time in the evenings (especially cellphone screens without night mode or blue light night filter); 6) regular exercise (preferably in the morning or afternoon); and 7) caffeinated beverages limited to the morning or early afternoon^^6^,^21^,^31^,^32^,^33^^. Moreover, we suggest that HCPs consider asking and guiding individuals with diabetes, as well as wider population and especially those with NCDs or in higher risk groups for severe COVID-19, about their sleep quality and routine.

In conclusion, we believe that there is ample evidence that strategies to keep the circadian rhythms, especially sleep-wake cycle, synchronized during the COVID-19 pandemic - improving sleep length and quality - can have significant effects on regulating the glycemia of individuals with diabetes, improving their immune response, well-being and, as a consequence, reducing the risk for severe consequences and mortality associated with SARS-CoV-2 infection.
